# Psoas Muscle Index Can Be Used to Predict Long-Term Mortality in Young Male Patients With Acute-on-Chronic Liver Failure

**DOI:** 10.3389/fnut.2022.811826

**Published:** 2022-02-18

**Authors:** Manman Xu, Tongzeng Li, Ming Kong, Nan Geng, Wenyan Song, Guanya Guo, Zhongping Duan, Ying Han, Yu Chen

**Affiliations:** ^1^Fourth Department of Liver Disease (Difficult & Complicated Liver Diseases and Artificial Liver Center), Beijing You'an Hospital, Capital Medical University, Beijing, China; ^2^Beijing Municipal Key Laboratory of Liver Failure and Artificial Liver Treatment Research, Beijing, China; ^3^Department of Infection Disease, Beijing You'an Hospital, Capital Medical University, Beijing, China; ^4^Department of Radiology, Beijing You'an Hospital, Capital Medical University, Beijing, China; ^5^Xijing Hospital of Digestive Diseases, Air Force Medical University (Fourth Military Medical University), Xian, China

**Keywords:** psoas muscle index, acute-on-chronic liver failure, long-term outcome, male, prognosis

## Abstract

**Objective:**

The use of psoas muscle index (PMI) in acute-on-chronic liver failure (ACLF) has not been reported, and the aim of this study was to evaluate the predictive value of PMI for the prognosis of patients with ACLF.

**Methods:**

In this study, male ACLF patients who underwent abdominal CT between 2015 and 2019 in our center were included to analyze the association between PMI and 1-year mortality in male ACLF patients, and subgroup analyses were performed according to age stratification (≤ 40 and >40 years).

**Results:**

We included 116 male patients with confirmed ACLF, with a mean PMI of 5.98 ± 1.68 cm^2^/m^2^ and a 1-year mortality of 51.7% (60). Univariate COX regression analysis showed that PMI was a protective factor [hazard ratio (HR), 0.851, 95%CI: 0.734–0.987] for 1-year mortality in male patients with ACLF. Nevertheless, multivariate analysis did not find an independent relationship between PMI and 1-year mortality. Subgroup analysis by age found that adjusted for MELD score, PMI was independently associated with 1-year mortality in young (age ≤ 40 years) male patients with ACLF (HR 0.689, 95% CI: 0.496–0.958). While no effect of PMI on 1-year mortality in non-young (age > 40 years) male ACLF patients was found. Correlation analysis found that there was no significant correlation between PMI and age in young (age ≤ 40 years) male ACLF patients, but, PMI decreased with age (*r* = −0.246, *P* < 0.05) in non-young (age > 40 years) male ACLF patients.

**Conclusion:**

PMI was found to be associated with 1-year mortality in male ACLF patients, especially in patients younger than 40 years, PMI predict 1-year mortality independent of MELD score.

## Introduction

The evaluation of nutritional status of patients with chronic liver disease by muscle mass and muscle function is receiving more and more attention. Sarcopenia is a major feature of malnutrition in patients with liver disease and is an important indicator affecting the prognosis of patients with end-stage liver disease (ESLD) (1). Numerous studies (2–5) have used the skeletal muscle index at the third lumbar vertebrae (L3-SMI) to determine sarcopenia, to further evaluate the association between sarcopenia and the prognosis of patients with chronic liver disease (CLD), and also some studies have evaluated the impact of the psoas muscle index (PMI) on the prognosis of CLD (6, 7). Studies (8–11) have shown that sarcopenia can be valuable as a predictor of disease progression, complications of cirrhosis such as the incidence of hepatic encephalopathy, mortality of cirrhotic patients, long-term outcome after liver transplantation, and outcome of patients with HCC.

Acute-on-chronic liver failure (ACLF) is a clinical syndrome manifested by acute liver decompensation in chronic liver disease (12) with rapid disease progression and high case fatality rate, and active exploration of indicators determining prognosis is valuable to guide treatment. This research team evaluated the predictive value of L3-SMI and sarcopenia for 90 day mortality in ACLF patients and found that sarcopenia had limited predictive value for the prognosis of ACLF (13) due to the heterogeneity of cut-off values for L3-SMI in diagnosing sarcopenia. Study (14) has shown that PMI is positively correlated with L3-SMI and is able to predict long-term prognosis in patients with cirrhosis (6).

As far as we can determine, there is a paucity of data exploring exploring the relationship between PMI and long-term outcomes in ACLF patients. Overall, this study aimed (1) to analyze the relationship between PMI and 1-year mortality in male ACLF patients and (2) to elucidate the effect of PMI on 1-year mortality risk in male ACLF patients in different age subgroups.

## Methods

### Patients

Patients with ACLF aged ≥18 years, who were hospitalized in Beijing You'an Hospital between January 2015 to June 2019, were retrospectively enrolled for the current study. Patients who met the following criteria were included: (1) 18 years of age or older; (2) underwent abdominal CT within 2 weeks of hospitalization; (3) diagnosed with ACLF according to the relevant diagnostic criteria of the Asian Pacific Association for the Study of the Liver (APASL) (15), manifested by jaundice (total bilirubin [TB] ≥ 5mg/dL) and coagulation dysfunction (international normalized ratio[INR] ≥ 1.5), and complicated within 4 weeks by ascites and/or hepatic encephalopathy (HE).

The exclusion criteria were as follows: (1) complicated by hepatocellular carcinoma (HCC) or other malignant tumors; (2) end stage disease of extrahepatic organs, such as respiratory failure or heart failure; (3) complicated by other consumptive diseases, such as tuberculosis or hyperthyroidism; (4) patients with neuromuscular diseases and those who were long-term bedridden; and (5) patients who had undergone long-term corticosteroid treatment.

All patient data were retrieved from electronic medical records. Follow-up was documented for 360 days after admission. The study procedures were approved by the Ethics Committees of Beijing You'an Hospital. As this was a retrospective study, informed consent was waived.

### Clinical Data

Clinical and laboratory results during the patients' hospitalization were collected, including sex, age, height, body mass, and complications, such as ascites and hepatic encephalopathy. Laboratory data were also collected at the diagnosis of ACLF, including routine blood tests, liver function, renal function, electrolytes and coagulation related indices.Information on liver transplantation and death were also collected for all enrolled patients and transplant-free survival/mortality was estimated for all enrolled patients at 360 days after enrollment or ACLF diagnosis. The patients' Model End-Stage Liver Disease (MELD) scores were calculated.

ACLF patients often have body fluid retention such as edema and ascites. In this study, the dry weight of ACLF patients with body fluid retention was calculated and corrected according to the clinical severity of ascites minus a certain amount of body weight (16) (mild 5%, moderate 10%, severe 15%, and 5% if there was peripheral edema). The body mass index (BMI) was calculated according to the following formula: BMI = dry weight (kg) / height squared (m^2^), and a BMI ≥ 25 kg/m^2^ was recognized as obesity in accordance with the BMI assessment criteria in China.

### Assessment of Psoas Muscle Index

**Figure 1:**
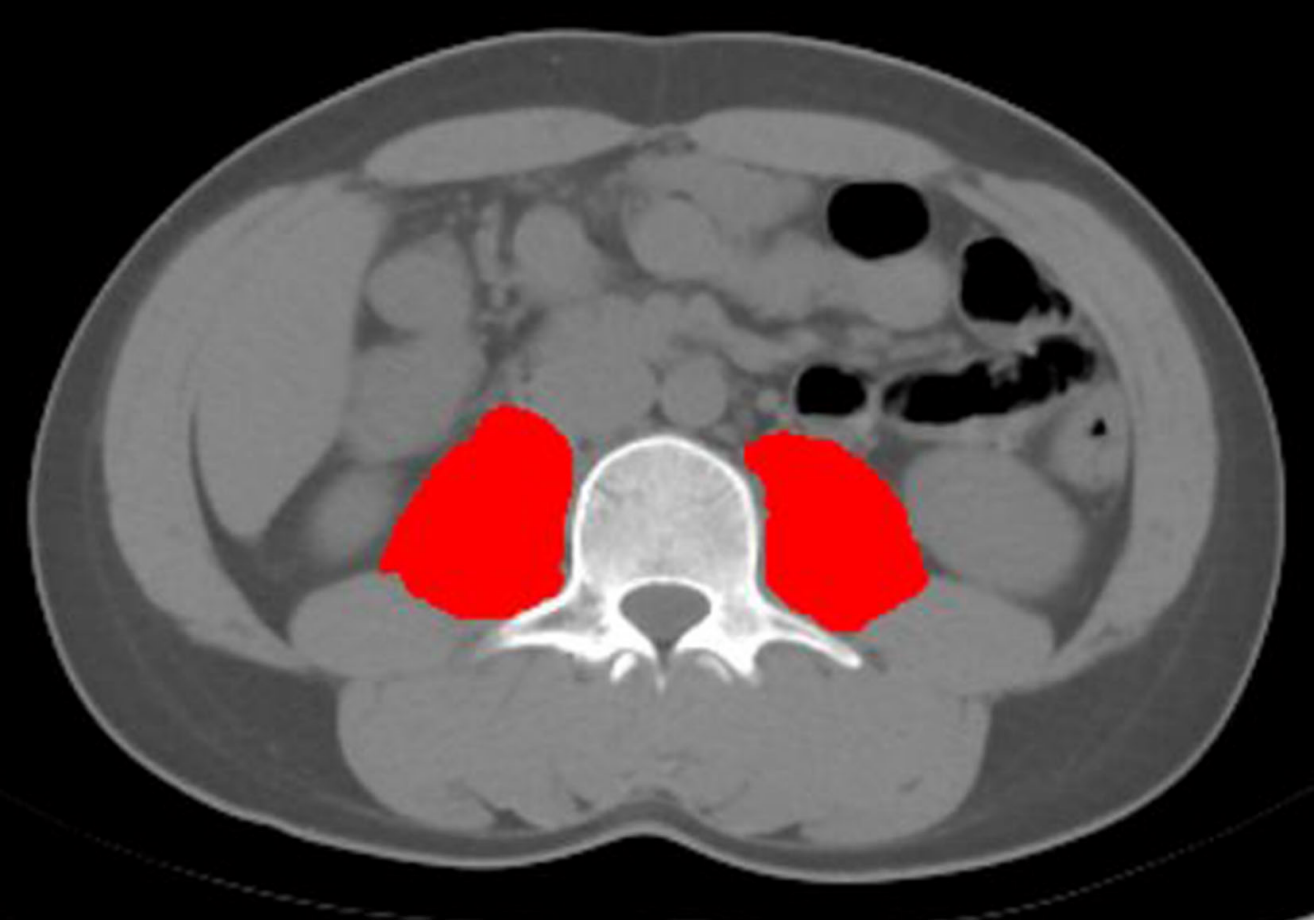
The psoas muscle mass area at the level of L3 vertebra.

Abdominal CT examinations were performed in all patients within 2 weeks of diagnosis of ACLF. CT scanning was performed with the LightSpeed VCT CT 64 scanner, USA. Psoas muscle area (cm^2^): the cross-sectional area (cm^2^) of the right and left psoas muscle at the third lumbar level (L3) on CT imaging estimates human skeletal muscle content (Figure 1). The psoas muscle area of the L3 cross-section was evaluated by two imaging physicians independently. When disagreement occurred, a third physician was involved and an agreement was reached.The psoas muscle index was calculated as the area of psoas muscle at the L3 level divided by the square of height (m^2^) to obtain the PMI (17).

### Statistical Analysis

Continuous variables are presented as mean ± standard deviation (SD) in the case of parametric data distribution or median (interquartile range (IQR)) in the case of non-parametric data distribution. Categorical variables are presented as a percentage. The Student's t-test was used for group comparisons of parametric data, while the Mann-Whitney-U test was used for non-parametric data. Group comparisons of categorical variables were performed using the χ2 test. Inter- and intra-observer agreement over the area of the psoas muscle were determined using the intraclass correlation coefficient (ICC). Clinical characteristics associated with mortality in ACLF patients were assessed using Multivariate Cox proportional hazards (PH) model,which were fitted with a stepwise method using significant baseline factors (candidate variables included PMI, complications and laboratory measurements, p <0.05) that had been prefiltered in univariate PH models to identify the independent relationship between PMI and mortality of patients with ACLF. Pearson correlation was used to analyze the correlation between age and PMI. *P*-values less than 0.05 were regarded as significant for 2-sided tests. All statistical analyses were performed with R × 64 4.0.3 (http://www.r-project.org/) GraphPad Prism Version 8.0 (GraphPad Software, La Jolla, CA, USA).

## Results

### Characteristics of the Patients

In the present study, we included 116 male patients with confirmed ACLF at a mean age of 43.64 (SD ± 10.43) years, including 61.0% (71) patients with cirrhosis. The most common etiology of ACLF was viral hepatitis (71.6%), followed by alcohol (17.2%). Overall, the median BMI in the study patients was 22.81 (20.48, 24.28) kg/m^2^, with 32.0% being obese (Table 1), and the median MELD scores was 24.34 (19.65, 26.95). For the measurement of psoas muscle area, a high intra-observer (ICC = 0.998, *p* < 0.001) and inter-observer agreement (ICC = 0.994, *p* < 0.001) were observed.The PMI of male patients with ACLF was 5.98 ± 1.68 cm^2^/m^2^.

**Table 1:** Baseline characteristics of surviving vs. non surviving patients in male acute-on-chronic liver failure.

**Variables**	**Total**	**Survivors**	**Non-survivors**	***p***
	**(*n* = 116)**	**(*n* = 60)**	**(*n* = 56)**
Age (years)	43.64 ± 10.43	40.88 ± 10.46	46.55 ± 9.66	0.003
Cirrhosis	71 (61)	27 (45)	44 (79)	<0.001
BMI (kg/m^2^)	22.81 (20.48, 24.28)	22.98 (20.66, 24.44)	22.63 (20.24, 24.19)	0.510
Obesity	37 (32)	22 (37)	15 (27)	0.346
ALB (g/L)	29.95 ± 5.54	30.06 ± 5.25	29.84 ± 5.88	0.831
TB (mg/dL)	21.85 (14.67, 29.18)	18.10 (11.00, 23.90)	26.50 (19.03, 32.18)	<0.001
INR	2.40 (2.10, 3.16)	2.26 (2.07, 2.66)	2.88 (2.12, 3.36)	0.006
CR (mg/dL)	0.70 (0.58, 0.83)	0.69 (0.56, 0.83)	0.71 (0.61, 0.82)	0.409
Na (mmol/L)	135.50 (131.70, 138.00)	136.90 (134.07, 138.83)	134.40 (130.15, 135.95)	<0.001
WBC (*10^9^/L)	6.97 (5.03, 9.80)	6.73 (5.20, 9.73)	7.32 (4.98, 9.50)	0.718
PLT (*10^9^/L)	96.00 (64.00, 149.00)	106.50 (74.00, 149.50)	82.00 (60.50, 139.00)	0.149
HGB (g/L)	121.15 ± 24.76	123.95 ± 23.62	118.15 ± 25.80	0.215
MELD score	24.34 (19.65, 26.95)	22.27 (18.48, 25.09)	26.04 (22.80, 28.58)	<0.001
Ascites	91 (78)	40 (67)	51 (91)	0.003
HE	27 (23)	8 (13)	19 (34)	0.016
PMI (cm^2^/m^2^)	5.98 ± 1.68	6.38 ± 1.77	5.56 ± 1.48	0.007

*Data were expressed as mean ± standard deviation, median (interquartile range), proportions or simple frequencies as appropriate. BMI, body mass index; ALB, albumin; TB, total bilirubin; INR, International normalized ratio; CR, Serum creatinine; Na, Serum sodium; WBC, White blood cell count; PLT, platelet; HGB, hemoglobin; MELD, model for end-stage liver disease; HE, Hepatic encephalopathy; PMI, psoas muscle index*.

### Baseline Characteristics of Surviving vs. Non-surviving Patients in Male ACLF

Of the 116 male ACLF patients, 60 (51.7%) patients were alive at 360 days (survivors), 51 patients died and 5 underwent liver transplantation (non survivors). Demographics, laboratory data, MELD scores and complications were compared between survival and non-survival patients with ACLF (Table 1). The mean age of patients in the male ACLF survival group was 40.88 ± 10.46 years, which was significantly lower than that of the non-survival group (46.55 ± 9.66 years) (*P* = 0.003); The proportion of cirrhotic patients in the non-survival group (79%) was significantly higher than that in the survival group (45%), and the difference was statistically significant.Total bilirubin, INR, MELD score were significantly higher and serum sodium level was significantly (*P* < 0.05) lower in the non-survival group compared to the survival group. The PMI of the surviving group patients was significantly lower than that of the non-surviving group patients [5.56 ± 1.48 cm^2^/m^2^ vs. 6.38 ± 1.77 cm^2^/m^2^, *P* = 0.007]. In addition, no significant difference was observed in the incidence of predisposing factors between patients with ACLF who survived and those who did not (Supplementary Table 1).

### PMI and Long-Term (360 Days) Mortality in Male Patients With ACLF

To clarify the independent relationship between PMI and long-term outcomes in male patients with ACLF, we performed univariate and multivariate Cox regression analyses of patients at 360 days of follow-up (Table 2). Univariate COX regression analysis showed that age, liver cirrhosis, total bilirubin, INR, lower serum sodium, MELD score, ascites and HE were risk factors for 360 day mortality in male patients with ACLF, while PMI was its protective factor (hazard ratio (HR), 0.851, 95%CI: 0.734–0.987) (*P* < 0.05).

**Table 2:** Univariate and multivariate COX regression models for male patients with ACLF.

	**Univariate**		**Multivariate**	
**Variables**	**HR (95% CI for HR)**	**p.value**	**HR (95% CI for HR)**	**p.value**
Age (years)	1.040 (1.010–1.060)	0.004		
Cirrhosis	2.820 (1.490–5.350)	0.002	2.746 (1.229–6.136)	0.014
BMI (kg/m^2^)	0.983 (0.918–1.050)	0.615		
Obesity	0.734 (0.406–1.330)	0.306		
ALB (g/L)	1.000 (0.956–1.050)	0.915		
TB (mg/dL)	1.050 (1.030–1.070)	0.000	1.041 (1.016–1.067)	0.001
INR	2.390 (1.570–3.630)	0.000	2.800 (1.798–4.363)	0.000
CR (mg/dL)	1.160 (0.642–2.110)	0.616		
Na (mmol/L)	0.912 (0.866–0.960)	0.000		
WBC (*10^9^/L)	0.987 (0.949–1.030)	0.533		
PLT (*10^9^/L)	0.997 (0.993–1.000)	0.191		
HGB (g/L)	0.992 (0.982–1.000)	0.163		
MELD score	1.100 (1.040–1.160)	0.000		
Ascites	3.630 (1.450–9.110)	0.006	4.902 (1.152–20.858)	0.031
HE	1.840 (1.060–3.210)	0.031		
PMI (cm^2^/m^2^)	0.851 (0.734–0.987)	0.033		

*The multivariate logistic regression model was fited with a stepwise selection method using statistically baseline factors that had been screened in univariate analysis. BMI, body mass index; ALB, albumin; TB, total bilirubin; INR, International normalized ratio; CR, Serum creatinine; Na, Serum sodium; WBC, White blood cell count; PLT, platelet; HGB, hemoglobin; MELD, model for end-stage liver disease; HE, Hepatic encephalopathy; PMI, psoas muscle index*.

When the above variables were included in the multivariate COX regression model, it was found that cirrhosis (HR, 2.746, 95%CI: 1.229–6.136),TB (HR, 1.041, 95%CI: 1.016–1.067), INR (HR, 2.800, 95%CI: 1.798–4.363) and ascites (HR, 4.902, 95%CI: 1.152–20.858) were risk factors for 360 day mortality in male patients with ACLF (*P* < 0.05). Nevertheless, multivariate analysis did not find an independent relationship between PMI and 360 day mortality in male ACLF patients.

### PMI in Male ACLF Patients of Different Age Subgroups

**Figure 2:**
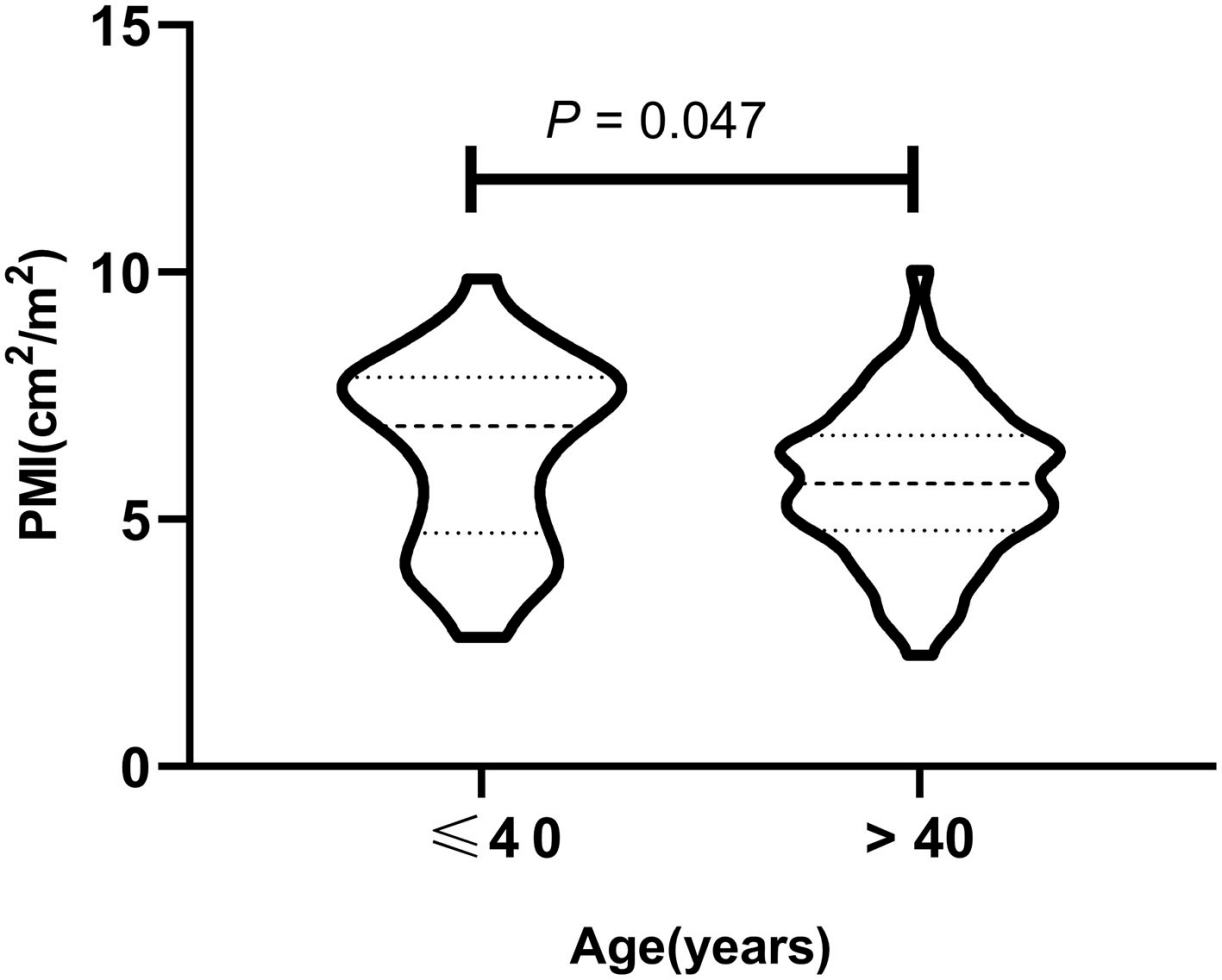
The psoas muscle index (PMI) in different age subgroups in male ACLF patients.

Skeletal muscle mass decreases gradually with age, and previous studies (17, 18) have shown that muscle mass decreases gradually from approximately age 40. Therefore, in this study, male ACLF patients were divided into two groups according to age: ≤ 40 and >40. The median PMI of the two groups were 6.89 (4.73–7.88) and 5.73 (4.78–6.70) cm^2^/m^2^, respectively, with significant differences (Figure 2) (*P* < 0.05). Univariate Cox regression analysis found an interaction effect of PMI ^*^ age ( ≤ 40 and >40 years) on 360 day mortality in male ACLF patients [0.647 (0.447–0.936), *P* = 0.021], so further subgroup analysis by age was performed in this study.

### PMI and Long-Term (360 Days) Mortality in Young (Age ≤ 40 Years) Male Patients With ACLF

A total of 41 male ACLF patients aged ≤ 40 years had a 360 day mortality rate of 29.3% (12), and the comparison between the survival and non survival groups was detailed in Supplementary Table 2. The median PMI of young male ACLF patients in the survival group was 7.34 (5.32, 7.97) cm^2^/m^2^, which was significantly higher than that of non-survival patients [5.47 (4.19, 6.46) cm^2^/m^2^, *P* = 0.014].

Following univariate Cox regression analysis of patients' clinical characteristics, TB, INR, MELD score, serum sodium and HE were significantly associated with mortality in patients with cirrhosis (Table 3), and low PMI is associated with increased 360 day mortality in young male ACLF patients [0.745 (0.557–0.997), *P* = 0.048]. Considering that there are too few dependent variables in our study, to avoid over fitting the model, according to the previous research results and clinical constraints, select MELD and PMI for multivariate analysis. The results showed that PMI was a factor affecting 360 day mortality in young male ACLF patients independently of MELD score (HR 0.689, 95%CI: 0.496–0.958).

**Table 3:** Univariate and multivariate Cox regression models in young (age ≤ 40 years) male ACLF patients.

	**Univariate**		**Multivariate**	
**Variables**	**HR (95% CI for HR)**	**p.value**	**HR (95% CI for HR)**	**p.value**
Age (years)	1.040 (0.932–1.160)	0.493		
Cirrhosis	3.360 (0.910–12.400)	0.069		
BMI (kg/m^2^)	0.977 (0.856–1.120)	0.730		
Obesity	0.927 (0.294–2.920)	0.897		
ALB (g/L)	0.942 (0.858–1.030)	0.205		
TB (mg/dL)	1.070 (1.010–1.130)	0.021		
INR	3.880 (1.610–9.380)	0.003		
CR (mg/dL)	5.460 (0.283–105.000)	0.261		
Na (mmol/L)	0.859 (0.783–0.941)	0.001		
WBC (*10^9^/L)	0.955 (0.859–1.060)	0.391		
PLT (*10^9^/L)	0.991 (0.980–1.000)	0.088		
HGB (g/L)	0.982 (0.961–1.000)	0.088		
MELD score	1.430 (1.190–1.720)	0.000	1.381 (1.137–1.676)	0.001
Ascites	0.025 (0.000–3.770)	0.150		
HE	3.700 (1.170–11.700)	0.026		
PMI (cm^2^/m^2^)	0.745 (0.557–0.997)	0.048	0.689 (0.496–0.958)	0.027

*The multivariate logistic regression model was fited with a stepwise selection method. Considering that there are too few dependent variables in our study, to avoid over fitting the model, according to the previous research results and clinical constraints, select MELD and PMI for multivariate analysis. BMI, body mass index; ALB, albumin; TB, total bilirubin; INR, International normalized ratio; CR, Serum creatinine; Na, Serum sodium; WBC, White blood cell count; PLT, platelet; HGB, hemoglobin; MELD, model for end-stage liver disease; HE, Hepatic encephalopathy; PMI, psoas muscle index*.

### PMI and Long-Term (360 Days) Mortality in Non-young (Age > 40 Years) Male Patients With ACLF

The 360 day mortality rate of 75 non-young male ACLF patients in this study was 58.67%, and the baseline characteristics of the patients between the survival and non-survival groups was detailed in Supplementary Table 3. The mean PMI of ACLF patients in survival group and non survival group were 5.99 ± 1.58 and 5.60 ± 1.50 cm^2^/m^2^, respectively, and the difference did not show statistical significance (*P* = 0.290). The univariate Cox regression analysis similarly did not find an independent relationship between PMI and the risk of 360 day mortality in non-young male ACLF patients. Multivariate Cox regression analysis identified TB and INR as independent risk factors for 360 mortality in non-young male ACLF patients (Table 4).

**Table 4:** Univariate and multivariate Cox regression models in non-young (age > 40 years) male ACLF patients.

	**Univariate**		**Multivariate**	
**Variables**	**HR (95% CI for HR)**	**p.value**	**HR (95% CI for HR)**	**p.value**
Age (years)	1.020 (0.973–1.060)	0.471		
Cirrhosis	2.260 (1.09–4.720)	0.029		
BMI (kg/m^2^)	1.010 (0.927–1.100)	0.852		
Obesity	0.826 (0.408–1.670)	0.595		
ALB (g/L)	1.030 (0.977–1.090)	0.271		
TB (mg/dL)	1.040 (1.020–1.070)	0.000	1.048 (1.022–1.074)	0.000
INR	1.950 (1.200–3.150)	0.007	1.921 (1.171–3.152)	0.010
CR (mg/dL)	0.893 (0.449–1.780)	0.747		
Na (mmol/L)	0.948 (0.887–1.010)	0.110		
WBC (*10^9^/L)	1.030 (0.972–1.080)	0.362		
PLT (*10^9^/L)	1.000 (0.995–1.000)	0.847		
HGB (g/L)	1.000 (0.987–1.010)	0.960		
MELD score	1.040 (0.991–1.100)	0.104		
Ascites	0.502 (0.198–1.276)	0.148		
HE	1.640 (0.845–3.200)	0.143		
PMI (cm^2^/m^2^)	0.946 (0.786–1.140)	0.555		

*The multivariate logistic regression model was fited with a stepwise selection method using statistically baseline factors that had been screened in univariate analysis. BMI, body mass index; ALB, albumin; TB, total bilirubin; INR, International normalized ratio; CR, Serum creatinine; Na, Serum sodium; WBC, White blood cell count; PLT, platelet; HGB, hemoglobin; MELD, model for end-stage liver disease; HE, Hepatic encephalopathy; PMI, psoas muscle index*.

### Correlation Between PMI and Age in Male ACLF Patients of Different Age Subgroups

**Figure 3:**
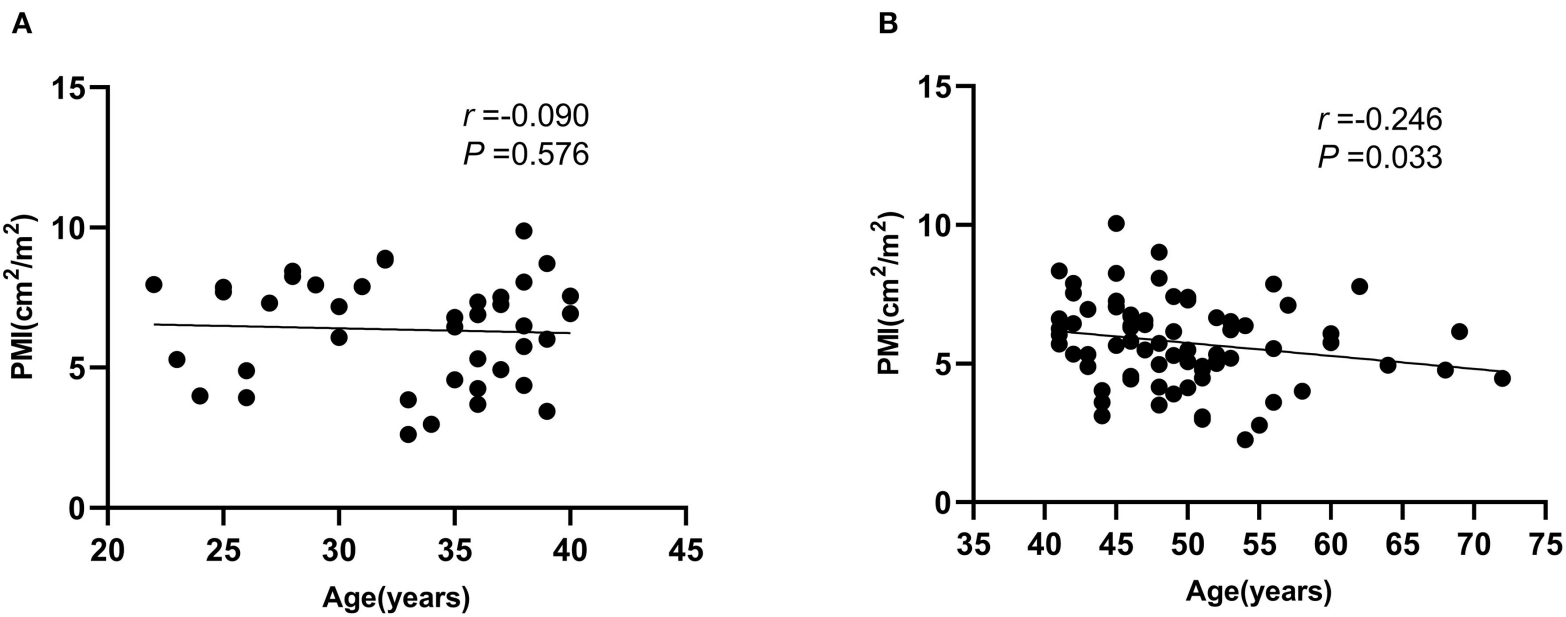
Correlation between psoas muscle index (PMI) and age in different age subgroups in male ACLF patients. **(A)** Age ≤ 40 years, **(B)** Age > 40 years.

To explore the reasons for the differences in the PMI effects on mortality in different age subgroups, we performed the correlation between PMI and age in different age subgroups (Figure 3), which showed that there was no significant correlation between PMI and age in young (age ≤ 40 years) male ACLF patients, however, there was a negative correlation between PMI and age in non-young (age > 40 years) male ACLF patients, which decreased with age (*P* < 0.05).

## Discussion

This study is the first to explore the effect of PMI on long-term outcomes (1 year) of ACLF patients, and the results found that PMI could distinguish 1-year survival from death in male ACLF patients. Found in different age subgroup analyses that for young male ACLF patients under 40 years old, PMI could predict 1-year mortality independently of MELD score, but for male ACLF patients over 40 years old, PMI was more affected by increasing age and had limited predictive value for 1-year mortality.

The predictive value of sarcopenia in chronic liver disease has been paid more and more attention (7, 19). Recently, a meta-analysis (20) confirmed that sarcopenia was highly and independently associated with higher risk of mortality in patients with cirrhosis, which was consistent with a large sample multicenter study in China (21). In addition, studies (22, 23) have reported that sarcopenia increases the risk of progression to ACLF and mortality in cirrhotic patients receiving transjugular intrahepatic portosystemic shunt. However, most studies have used SMI as a criterion to evaluate sarcopenia, and very few studies have evaluated the predictive prognostic value of PMI in chronic liver diseases. A multicenter study (24) that evaluated the prognostic impact of SMI vs. PMI in cirrhotic patients showed that SMI is a more complete and reliable measure than PMI, especially in male cirrhotic patients, and patients at high risk of mortality as determined by SMI were misclassified as low risk of mortality by the PMI cut-off value, so the investigators concluded that SMI should not be replaced by PMI. On the contrary, our scholars (6) developed a prognostic model that included the PMI through the long-term follow-up of cirrhotic patients, and the results showed that the PMI was an independent predictor of the 3-year mortality risk of cirrhotic patients and that the PMI was associated with the gait speed of the patients. The c-index of the predictive model that included the PMI was 0.792 (95% CI: 0.723–0.861) in men and 0.715 (95% CI: 0.637–0.793) in women, respectively, implying that a prediction model containing PMI can predict long-term mortality in cirrhotic patients with high efficiency. An additional study (25) evaluating the impact of PMI on outcomes after liver transplantation demonstrated that the 120 day survival rate after liver transplantation was significantly lower in the lower PMI preoperative group than in the higher PMI group, that there was a significant association between preoperative PMI and short-term postoperative outcomes, and that sarcopenia estimated by PMI could be used as a predictor of mortality risk after liver transplantation. Another study that used PMI to diagnose sarcopenia found that sarcopenia was associated with post-transplant infections, requirement for mechanical ventilation, intensive care (ICU) and hospital stay, and 1 year mortality in liver transplant recipients (26).

Currently, most of the reported predictive values of PMI in CLD have focused on patients undergoing liver transplantation, cirrhotic patients. The present study confirmed the value of PMI in predicting the long-term prognosis of ACLF, especially for young male ACLF patients, and showed a higher predictive value than SMI compared with the results of previous study (13), which may have several reasons: First, the SMI was based on the whole skeletal muscle area at the cross-section of the third lumbar body, while the PMI was based on the sum of the areas of the right and left psoas muscles at the third lumbar paraspinal body with less systematic error and better accuracy. Second, the predictive value of the PMI for prognosis in ACLF in men over 40 years of age was not found in this study, and for this part of the population, the decline in skeletal muscle mass was more influenced by increasing age, while the association with muscle wasting due to liver failure is not strong, and the specific mechanism still needs further validation in a large sample multicenter prospective study. A retrospective cohort study (27) of pediatric end-stage liver disease patients who underwent liver transplantation found that lower PMI were associated with higher reoperation rates and longer posttransplant hospital stays. It is thus speculated that the PMI has more value in judging prognosis in young patients with liver disease.

In addition to its retrospective design, there are some limitations in this study. First, only male ACLF patients were included in this study, and the sample size was small, especially in different age subgroups. Second, this study only evaluated the PMI at baseline in ACLF patients and did not evaluate the dynamic changes of PMI during the course of ACLF, so the effect of PMI on the prognosis of ACLF was not fully evaluated. However, the current study is the first to evaluate the value of PMI in ACLF and provides a reference for further research.Third, this study evaluated the long-term outcomes of patients with ACLF and only analyzed the impact of baseline data on outcomes and did not consider the impact of treatment regimen and patient compliance on outcomes. Finally, the mechanism of muscle mass wasting in ACLF patients was not elucidated in this study, which is our next focus.

In conclusion, in our study cohort, PMI was found to be associated with 1-year mortality in male ACLF patients, especially in patients younger than 40 years, PMI predict 1-year mortality independent of MELD score. The value of PMI in ACLF needs additional basic research and larger clinical studies to clarify.

## Data Availability Statement

The original contributions presented in the study are included in the article/Supplementary Material, further inquiries can be directed to the corresponding author/s.

## Ethics Statement

The studies involving human participants were reviewed and approved by the Ethics Committees of Beijing You'an Hospital. The Ethics Committee waived the requirement of written informed consent for participation.

## Author Contributions

YC: conceptualization and supervision. MX, TL, MK, GG, and YC: methodology, formal analysis, visualization, and writing—original draft. MX, TL, and WS: resource. MX, TL, YH, ZD, WS, and YC: data curation and writing—review and editing. All authors have made an intellectual contribution to the manuscript and approved the submission.

## Funding

This study was partly supported by Beijing Advanced Innovation Center for Big Data-Based Precision Medicine (PXM2021_014226_000026) and Capital's Funds for Health Improvement and Research (2021-1G-2181).

## Conflict of Interest

The authors declare that the research was conducted in the absence of any commercial or financial relationships that could be construed as a potential conflict of interest.

## Publisher's Note

All claims expressed in this article are solely those of the authors and do not necessarily represent those of their affiliated organizations, or those of the publisher, the editors and the reviewers. Any product that may be evaluated in this article, or claim that may be made by its manufacturer, is not guaranteed or endorsed by the publisher.
