# Supplementary material for: Psoas Muscle Index Can Be Used to Predict Long-Term Mortality in Young Male Patients With Acute-on-Chronic Liver Failure
Source: Front Nutr. 2022 Feb 18;9:811826. doi: 10.3389/fnut.2022.811826 (PMC8894235; doi:10.3389/fnut.2022.811826)
Supplement: Supplementary file 1 [file Table_1.DOCX]

Supplementary table 1.Association between 360-day outcome and predisposing factors in patients with ACLF.

| Variable | Total  (n = 116) | Survivors  (n = 60) | Non-survivors  (n = 56) | P value |
| --- | --- | --- | --- | --- |
| Predisposing factors, n (%) |  |  |  | 0.557 |
| Reactivation of HBV | 27(23.3) | 12（20.0） | 15（26.8） |  |
| Alcohol | 13(11.2) | 7（11.7） | 6（10.7） |  |
| Bacterial infection | 6(5.2) | 3（5.0） | 3（5.4） |  |
| Drugs or Poisons | 6(5.2) | 5（8.3） | 1（1.8） |  |
| Unclear | 64（55.2） | 33（55.0） | 31（55.4） |  |

Supplementary Table 2 Baseline characteristics of young (≤ 40 years) male patients with ACLF

| Variables | Total  (n = 41) | Survivors  (n = 29) | Non-survivors  (n = 12) | p |
| --- | --- | --- | --- | --- |
| Age(years) | 35.00 (28.00, 37.00) | 33.00 (28.00, 37.00) | 36.00 (29.25, 38.00) | 0.430 |
| Cirrhosis | 21 (51) | 12 (41) | 9 (75) | 0.106 |
| BMI(kg/m^2^) | 23.79 ± 4.63 | 24.01 ± 4.54 | 23.27 ± 5.02 | 0.667 |
| Obesity | 18 (44) | 13 (45) | 5 (42) | 1.000 |
| ALB(g/L) | 30.10 ± 5.63 | 30.96 ± 5.34 | 28.00 ± 5.99 | 0.154 |
| TB(mg/dL) | 21.80 ± 10.90 | 19.50 ± 9.90 | 27.18 ± 11.65 | 0.062 |
| INR | 2.42 (2.06, 3.22) | 2.23 (2.02, 2.78) | 3.22 (2.44, 3.43) | 0.030 |
| CR(mg/dL) | 0.62 (0.55, 0.74) | 0.62 (0.53, 0.74) | 0.63 (0.60, 0.75) | 0.282 |
| Na(mmol/L) | 136.60 (131.90, 138.70) | 137.90 (136.00, 139.60) | 130.90 (127.25, 135.20) | 0.001 |
| WBC(*10^9^/L) | 7.29 (5.56, 11.16) | 8.44 (5.56, 12.33) | 6.84 (5.80, 8.25) | 0.387 |
| PLT(*10^9^/L) | 119.22 ± 67.79 | 130.24 ± 66.02 | 92.60 ± 67.26 | 0.117 |
| HGB(g/L) | 126.24 ± 26.27 | 130.38 ± 21.22 | 116.25 ± 34.78 | 0.211 |
| MELD score | 22.65 ± 5.00 | 20.93 ± 3.94 | 26.82 ± 4.96 | 0.002 |
| Ascites | 29 (71) | 17 (59) | 12 (100) | 0.008 |
| HE | 12 (29) | 5 (17) | 7 (58) | 0.020 |
| PMI(cm^2^/m^2^) | 6.89 (4.89, 7.87) | 7.34 (5.32, 7.97) | 5.47 (4.19, 6.46) | 0.014 |

Data were expressed as mean ± standard deviation, median (interquartile range), proportions or simple frequencies as appropriate.

BMI, body mass index; ALB, albumin; TB, total bilirubin; INR, International normalized ratio;

CR, Serum creatinine; Na, Serum sodium; WBC, White blood cell count; PLT, platelet; HGB hemoglobin; MELD, model for end-stage liver disease; HE, Hepatic encephalopathy; PMI, psoas muscle index.

Supplementary Table 3 Baseline characteristics of young (> 40 years) male patients with ACLF

| Variables | Total  (n = 75) | Survivors  (n = 31) | Non-survivors  (n = 44) | p |
| --- | --- | --- | --- | --- |
| Age(years) | 48.00 (45.00, 52.50) | 49.00 (45.50, 52.00) | 48.00 (45.00, 53.25) | 0.792 |
| Cirrhosis | 50 (67) | 15 (48) | 35 (80) | 0.010 |
| BMI(kg/m^2^) | 22.46 (20.34, 23.90) | 22.59 (20.27, 24.09) | 22.44 (20.75, 23.88) | 0.916 |
| Obesity | 19 (25) | 9 (29) | 10 (23) | 0.727 |
| ALB(g/L) | 29.88 ± 5.53 | 29.19 ± 5.10 | 30.34 ± 5.82 | 0.373 |
| TB(mg/dL) | 23.13 ± 10.32 | 17.48 ± 7.27 | 27.17 ± 10.35 | < 0.001 |
| INR | 2.40 (2.12, 3.02) | 2.26 (2.12, 2.51) | 2.78 (2.12, 3.34) | 0.095 |
| CR(mg/dL) | 0.74 (0.60, 0.87) | 0.78 (0.56, 0.95) | 0.72 (0.61, 0.84) | 0.640 |
| Na(mmol/L) | 134.85 (131.62, 137.20) | 136.20 (133.60, 138.15) | 134.70 (131.05, 136.05) | 0.073 |
| WBC(*10^9^/L) | 6.79 (4.96, 9.03) | 6.03 (5.14, 8.20) | 7.38 (4.91, 10.39) | 0.159 |
| PLT(*10^9^/L) | 93.00 (64.50, 124.75) | 100.00 (67.50, 116.50) | 86.00 (64.00, 131.50) | 0.705 |
| HGB(g/L) | 118.29 ± 23.56 | 117.73 ± 24.49 | 118.69 ± 23.18 | 0.868 |
| MELD score | 24.82 (20.16, 27.27) | 23.40 (18.83, 25.85) | 25.96 (22.44, 28.26) | 0.099 |
| Ascites | 62 (83) | 23 (74) | 39 (89) | 0.188 |
| HE | 15 (20) | 3 (10) | 12 (27) | 0.113 |
| PMI(cm^2^/m^2^) | 5.76 ± 1.54 | 5.99 ± 1.58 | 5.60 ± 1.50 | 0.290 |

Data were expressed as mean ± standard deviation, median (interquartile range), proportions or simple frequencies as appropriate.

BMI, body mass index; ALB, albumin; TB, total bilirubin; INR, International normalized ratio;

CR, Serum creatinine; Na, Serum sodium; WBC, White blood cell count; PLT, platelet; HGB hemoglobin; MELD, model for end-stage liver disease; HE, Hepatic encephalopathy; PMI, psoas muscle index.
